# Сomplete genome sequence of *Saccharomyces cerevisiae* I-118, a wine strain isolated from Rkatsiteli grapes in Uzbekistan

**DOI:** 10.1128/mra.01229-25

**Published:** 2025-12-19

**Authors:** Alexey V. Beletsky, Egor A. Vasyagin, Maxim Yu. Shalamitskiy, Elena V. Ivanova, Nikolai V. Ravin, Andrey V. Mardanov

**Affiliations:** 1 Institute of Bioengineering, Research Center of Biotechnology of the Russian Academy of Sciences 96707, Moscow, Russia; 2 All-Russian National Research Institute of Viticulture and Winemaking “Magarach” of the National Research Centre “Kurchatov Institute”, Yalta, Russia; Rochester Institute of Technology, Rochester, New York, USA

**Keywords:** *Saccharomyces cerevisiae*, wine strain, complete genome

## Abstract

We report the complete genome sequence of the wine yeast strain *Saccharomyces cerevisiae* I-118. It was isolated from yeast sediment formed after spontaneous fermentation of Rkatsiteli grapes and is used to produce white wines. Genomic data provide the basis for linking the genotype of this strain with its oenological properties.

## ANNOUNCEMENT

For millennia, the yeast *Saccharomyces cerevisiae* has been used to produce fermented foods (1). This yeast is essential for winemaking, producing one of the most popular beverages worldwide (2). During the winemaking process, *S. cerevisiae* ferments the sugars present in grape juice into alcohol (3). Fermentation also generates a range of aromatic compounds, including higher alcohols, aldehydes, volatile acids, esters, ketones, and others (4), which impart a unique aromatic profile to the wine. During fermentation, factors, such as temperature, the choice of yeast strain, and the duration of fermentation, exert a substantial influence on the wine’s quality and organoleptic properties (5). The search for novel yeast strains with diverse metabolic capabilities for the synthesis of secondary metabolites and varying technological parameters is key to advancing winemaking technology. Therefore, the isolation and characterization of new yeast strains possessing distinctive regional characteristics remain a relevant and ongoing objective for the wine industry. The aim of our study was to sequence the complete genome of the *S. cerevisiae* I-118 strain from the Magarach collection of winemaking microorganisms. The strain was isolated from the Rkatsiteli grape variety in experimental vineyards of the Central Asian branch of the Magarach Institute in Uzbekistan in 1948. Yeast sediment samples were collected at the end of spontaneous fermentation, homogenized, serially diluted, and plated on 2% grape must agar. After incubation at 25°C for three days, randomly selected colonies were purified. The strain is preferred for the production of white table and sparkling wines (6).

To isolate genomic DNA, the strain was cultured for two days in YPD medium at 28°C, followed by extraction using the DNeasy PowerSoil DNA Isolation Kit (Qiagen, Germany).

For genome sequencing, we employed a hybrid sequencing approach, integrating the Illumina and Oxford Nanopore platforms. The DNA library for Illumina sequencing was prepared with the NEBNext Ultra II DNA Library Prep Kit (New England Biolabs, USA). Illumina sequencing was performed on a MiSeq instrument (Illumina, USA), producing 2,551,019 pairs of 300 nt reads. Adapter sequences were found and removed using Cutadapt v.4.0 with default parameters (7). Overlapping paired-end reads were merged using FLASH v.1.2.11 with the “-M 300” parameter set (8). Low-quality bases at 3′ ends were removed using Sickle v.1.33 (https://github.com/najoshi/sickle) with a quality threshold of Q30. The genomic DNA was additionally sequenced on a MinION (Oxford Nanopore Technologies, UK). The genomic library was prepared from non-sheared DNA without size selection using the Ligation Sequencing gDNA Kit (SQK-LSK109) and sequenced on FLO-MIN110 flow cells. The MinION run produced 345,116 reads with an N50 length of 9,659 nucleotides. Guppy v.5.0.7 (Oxford Nanopore Technologies) was used for the basecalling and quality filtering; parameters were automatically selected based on the flow cell and kit. Complete chromosomes were *de novo* assembled from MinION reads using Flye v.2.8.1, with the parameters “--nano-raw and –iterations 2” specified (9). Consensus accuracy of the chromosomes was improved by Illumina short reads using the POLCA tool of MaSuRCA v.4.1.4 (10). Genome statistics of the resulting complete genome are described in Table 1.

**TABLE 1:** Genome sequencing statistics

Parameter	Value
Illumina sequencing	
Read pair number	2,551,019
Total bases	1,320,422,001
Nanopore sequencing	
Read number	345,116
Total bases	2,368,130,350
Genome assembly	
Total base	12,030,035
Chromosome length (nucleotides)	
I	203,290
II	799,357
III	308,002
IV	975,404
V	552,952
VI	250,085
VII	1,564,366
VIII	534,789
IX	425,954
X	727,006
XI	675,926
XII	1,281,890
XIII	906,136
XIV	792,621
XV	1,031,908
XVI	919,195
Mitochondrion length (nucleotides)	81,154
GC content (%)	38.30
Protein-coding genes	5,348
tRNA genes	300
Non-coding DNA (%)	31.58
Genome coverage	226×
Copies of rRNA gene cluster	About 31
Genome assembly assessment	
Complete BUSCOs	99.30%
Fragmented BUSCOs	0.00%
Missing BUSCOs	0.70%

Model strain *S. cerevisiae* S288C was chosen to predict gene structures using Augustus v.3.4.0 (11). Predictions were improved by integrating hints into the Augustus pipeline. Hints were obtained by constructing splice-aware alignments of *S. cerevisiae* strain S288C genes to the chromosomes using BLAT v.34. The “--genemodel=complete –noInFrameStop=true” parameters for Augustus were specified (12). Genome completeness was evaluated using BUSCO v.5.7.1 (13) with the *Saccharomycetes* database. Default parameters were used for all software unless otherwise noted.

The complete genome of strain I-118 contributes to comparative genomic studies of *S. cerevisiae* wine strains.

## Data Availability

Raw reads have been deposited in the NCBI Sequence Read Archive under the accession numbers SRR35743130 (Illumina) and SRR35743129 (MinION). The assembled genome has been deposited in DDBJ/ENA/GenBank under accession numbers AP044972 (chromosome I), AP044973 (chromosome II), AP044974 (chromosome III), AP044975 (chromosome IV), AP044976 (chromosome V), AP044977 (chromosome VI), AP044978 (chromosome VII), AP044979 (chromosome VIII), AP044980 (chromosome IX), AP044981 (chromosome X), AP044982 (chromosome XI), AP044983 (chromosome XII), AP044984 (chromosome XIII), AP044985 (chromosome XIV), AP044986 (chromosome XV), AP044987 (chromosome XVI), AP044988 (mithochondrion). The version described in this paper is the first version.
